# Utilizing network pharmacology and other tools to examine active components and mechanism of action of *Magnolia officinalis rheum rhabarbarum* decoction in treating *Streptococcus pyogenes* skin infections

**DOI:** 10.1186/s40643-025-00933-1

**Published:** 2025-08-26

**Authors:** Yuanhao Wang, Xinrui Wang, Xueying Zhang, Mengyi Pan, Mingyang Sun, Zhiguo chen, Yingli Song

**Affiliations:** 1 School of Basic Medical Sciences, Harbin Medical University, No. 157, Health Care Road, Nangang District, Harbin City, Heilongjiang Province China; 2 The Second Affiliated Hospital of Harbin Medical University, No.246 Xuefu Road, Nangang District, Harbin City, Heilongjiang Province China

**Keywords:** Network pharmacology, Drug discovery, Molecular docking, *Streptococcus pyogenes*

## Abstract

Infections caused by *Streptococcus pyogenes* and the growing threat of antibiotic resistance pose significant global health challenges. This study investigates the antibacterial properties of *Magnolia officinalis Rheum rhabarbarum* Decoction against *Streptococcus pyogenes* skin infections. By combining UHPLC-MS/MS, network pharmacology, and molecular docking techniques, we identified eight bioactive compounds in the formulation and explored their potential interactions with *Streptococcus pyogenes*-related targets. Our analysis revealed that compounds such as Sinensetin, Nobiletin, and (+)-Magnoflorine regulate immune pathways (IL-17, TNF), inhibit the production of inflammatory factors, and disrupt bacterial membranes and metabolic processes, achieving dual antibacterial and anti-inflammatory effects. In vitro experiments showed that the decoction exhibited a minimum inhibitory concentration (MIC) of 20 mg/mL against *Streptococcus pyogenes*, significantly reducing the secretion of pro-inflammatory factors such as IL-1α, IL-6, IL-36, and TNF-α. These results suggest that *Magnolia officinalis Rheum rhabarbarum* Decoction offers a promising multi-target strategy for treating drug-resistant *Streptococcus pyogenes* infections and may serve as a potential alternative to traditional antibiotics.

**Graphical abstract:**

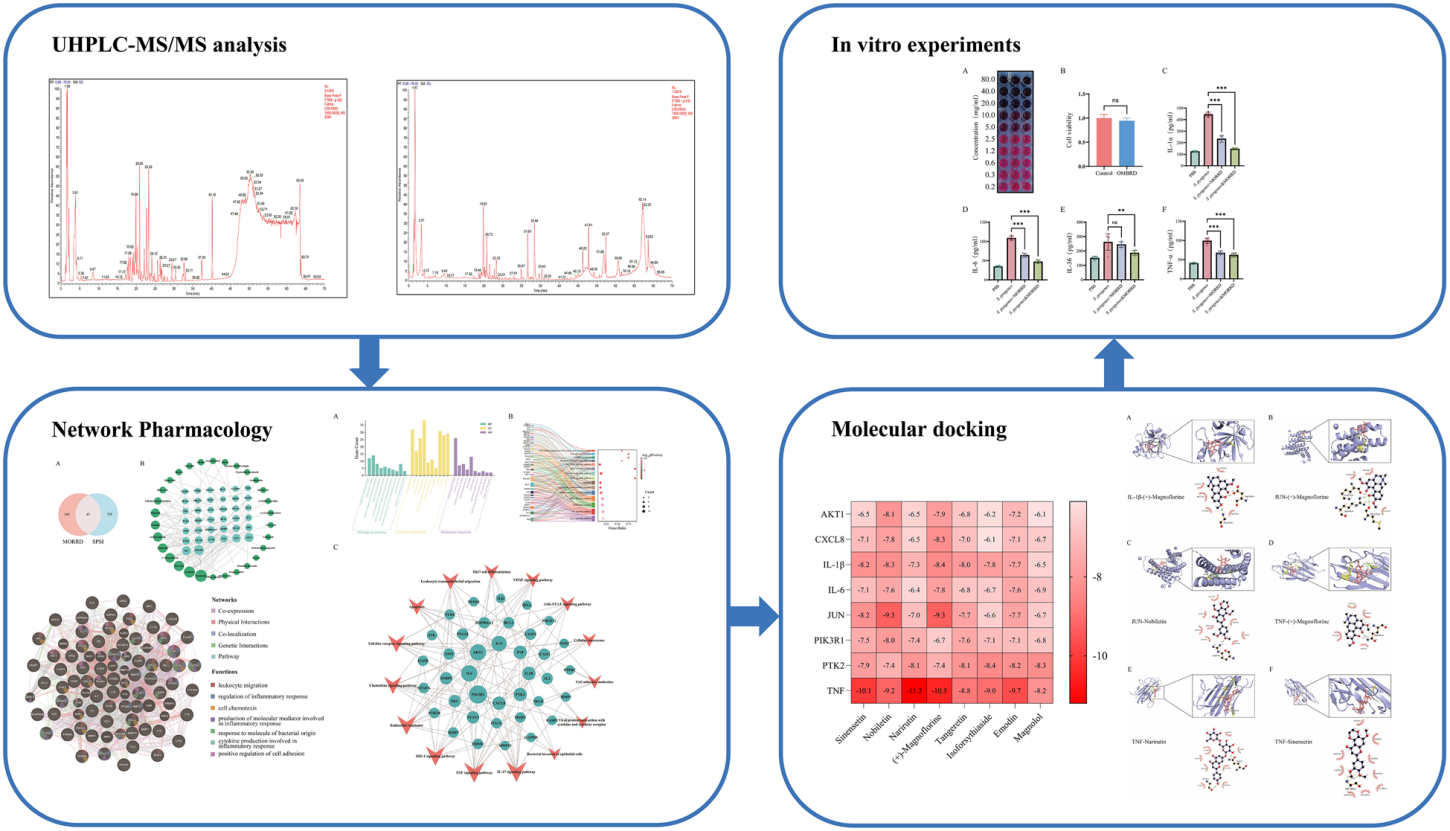

**Supplementary Information:**

The online version contains supplementary material available at 10.1186/s40643-025-00933-1.

## Introduction

The largest organ in the human body is the skin, which covers the entire body. Maintaining adequate hydration is essential for preserving internal homeostasis, shielding the body from normal wear and tear, regulating body temperature, and enhancing sensory perception (Bae et al. 2022; Baker et al. 2023). Under normal conditions, the many microorganisms that invade the human skin are harmless. However, specific bacteria experience dysbiosis and attack the host when the host’s immunity deteriorates. We call these bacteria opportunistic pathogens. *Streptococcus pyogenes*(*S*. *pyogenes*) is a common human commensal bacterium that represents such infections and continues to pose a serious hazard to public health worldwide (Howden et al. 2023). The World Health Organization (WHO) noted at the end of 2022 that invasive infections and scarlet fever had increased significantly in some affluent nations, with children being disproportionately affected (Wang et al. 2023).

**Fig. 1:**
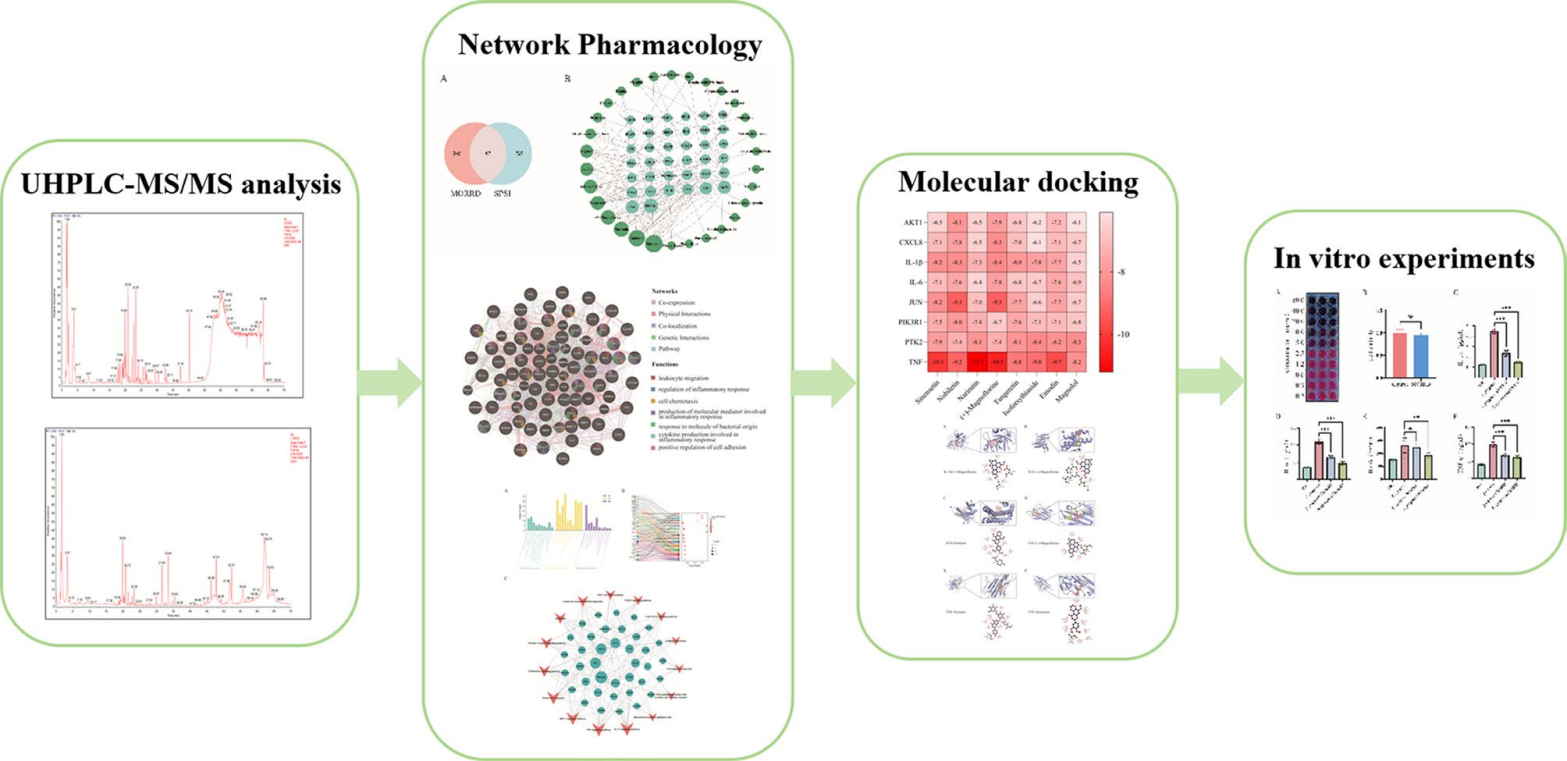
Graphical summary

*S*. *pyogenes*, also known as Group A Streptococci (GAS), is an obligate human pathogen associated with a wide range of illnesses. The spectrum encompasses superficial infections, such as streptococcal pharyngitis and skin infections like impetigo and cellulitis, as well as life-threatening invasive or toxin-mediated diseases, including necrotizing fasciitis, streptococcal toxic shock syndrome, scarlet fever, and septic shock (Bae et al. 2022).

Among various bacteria that cause skin infections, *S*. *pyogenes*, an obligate human pathogen, is particularly concerning due to its ability to cause a wide range of diseases, from superficial impetigo to deep tissue infections such as necrotizing fasciitis. *S*. *pyogenes* skin infection(SPSI), which encompass severe invasive infections of the dermis and deeper tissues like cellulitis and necrotizing fasciitis, as well as superficial epidermal infections like impetigo, are especially alarming. Treating invasive *S. pyogenes* infections is challenging because of the bacterium’s ability to form biofilms and produce various toxins, even though it is generally responsive to several antibiotics, including β-lactams. Biofilms, structured bacterial populations embedded in extracellular polymeric materials, enhance the bacteria’s resistance to antibiotic treatment. Numerous membrane-damaging hemolysins, immune-modulating superantigens, plasminogen activators, host cell adhesins, complement regulatory proteins, specific and nonspecific proteases, and many other degradative enzymes produced by *S. pyogenes* significantly contribute to the destruction of host tissue (Alves-Barroco et al. 2020; Zou et al. 2024).

The rise of antibiotic resistance poses a significant threat to global healthcare systems and suggests the possibility of a post-antibiotic era. Antibiotic overuse and misuse, along with environmental reservoirs of antibiotic-resistant bacteria, have contributed to the increasing rates of resistance (Collaborators 2022). The slowdown in the development of new antimicrobials and dependence on current antibiotics, coupled with inadequate antibiotic stewardship, has worsened the evolution of resistance (Baker et al. 2018). There is an urgent need for agents that can effectively combat resistant pathogens.

The extensive use of antibiotics has accelerated the development of resistance, making the treatment of many common infectious diseases increasingly challenging and heightening the risk of encountering untreatable infections. In contrast to single-component antibiotics, Traditional Chinese Medicine (TCM) functions through multiple pathways and targets, which reduces the likelihood of developing bacterial resistance. The benefits of TCM in treating bacterial infections, especially antibiotic-resistant strains, have received considerable attention. *Magnolia officinalis Rheum rhabarbarum* Decoction (MORRD) is a traditional Chinese medicinal formula documented in Jin Kui Yao Lue, historically used to alleviate symptoms such as chest fullness and abdominal pain. This formula contains *Magnolia officinalis* (MO), *Rheum rhabarbarum* L. (RR), and *Aurantii Fructus Immaturus* (AF), which are decocted in water and taken orally. The MO demonstrates a range of pharmacological activities, encompassing antibacterial, antiviral, and antitumor effects are observed in compounds like magnolol and honokiol found in MO, which alter the structure and function of bacterial cell membranes, thereby inhibiting bacterial growth and survival. These components exhibit inhibitory effects on various Gram-positive bacteria and certain Gram-negative bacteria (Sun et al. 2023; Zheng et al. 2024). The primary antibacterial components of RR are anthraquinones, including rhein, emodin, and aloe-emodin. These compounds inhibit various pathogenic bacteria by disrupting bacterial membranes, suppressing nucleic acid and protein synthesis, and interfering with carbohydrate metabolism (Zheng et al. 2021). AF contains hesperidin and related flavonoids, which demonstrate specific antibacterial activities.

The advancement of multi-omics technologies, artificial intelligence, and Big Data analysis has led to the rapid development and widespread application of network pharmacology. Network pharmacology aligns with the comprehensive attributes of TCM and represents a new field with pioneering methodologies. Researchers are increasingly using network pharmacology to examine the active compounds, targets, and molecular mechanisms of TCM. Identifying targets and mechanisms in TCM is a central objective of TCM network pharmacology. This approach, through network construction, analysis, and validation, elucidates the multi-target and multi-pathway action patterns of TCM, clarifying the pharmacodynamic targets and molecular mechanisms that underlie its efficacy. The multi-component and multi-target characteristics of TCM formulas make network pharmacology’s holistic and systemic research approach particularly advantageous for investigating the mechanisms of TCM compatibility. This approach also provides theoretical support for understanding the principles underlying TCM formulas (Wu et al. 2022; Zhou et al. 2023).

## Results

### Active components and targets of MORRD

**Fig. 2:**
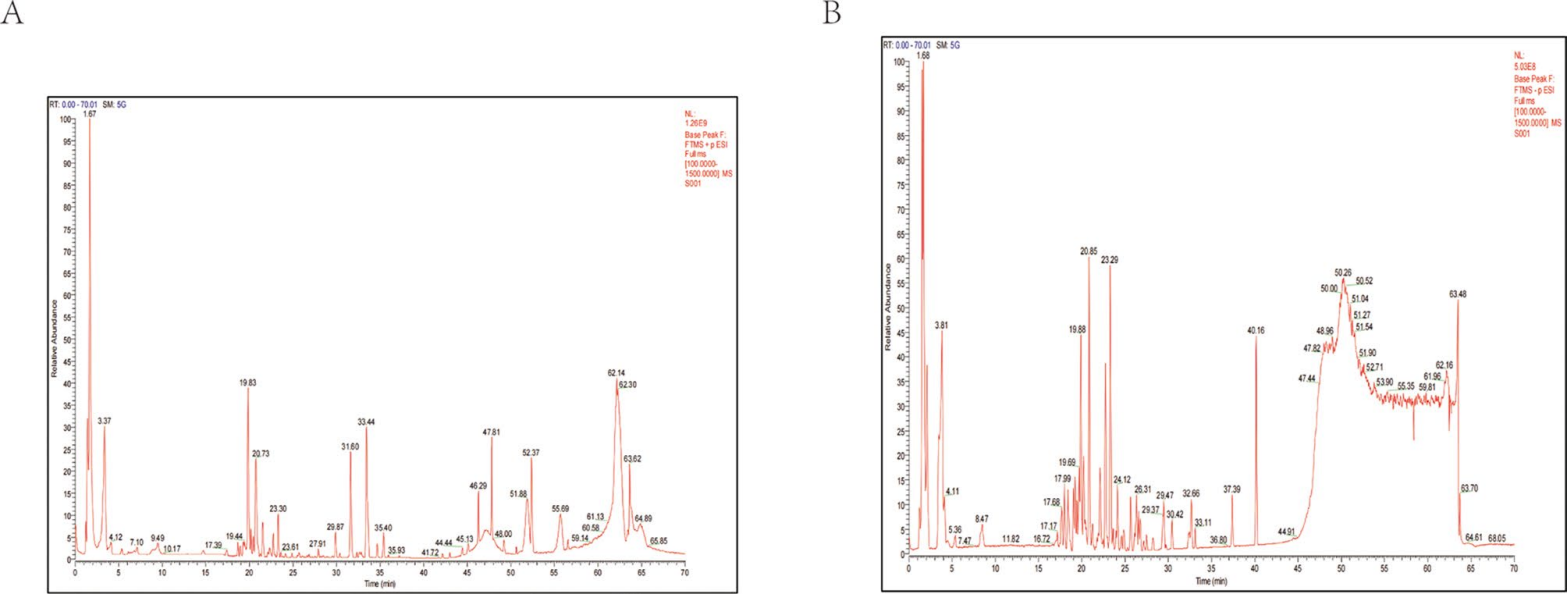
TIC of the sample extracts under positive and negative ion modes. **A**. TIC of the sample extract in positive ion mode. **B**. TIC of the sample extract in negative ion mode

Using a Thermo QE Plus liquid chromatography system in conjunction with a tandem high-resolution mass spectrometry system, the chemical components of MORRD were examined. Compound Discoverer software was utilized to process the data. Both positive and negative ion modes were employed to create Total Ion Chromatograms (TIC) for each sample (Fig. 2A).

MORRD identified and detailed a total of 120 intrinsic chemical components (Supplementary Data, Table 1). The bioactivities of the top 30 compounds are summarized in Table 1, which ranks the discovered compounds according to the intensity of their mass spectrometry response. The SwissTargetPrediction database was updated with the SMILES strings of the active chemicals sourced from the PubChem database. After removing duplicate targets, a total of 413 unique potential targets were identified (Fig. 2B).

**Table 1:** Bioactivity information of the top 30 ranked compounds

Name	Molecular formula	Relative content (%)	PubChem CID
Stachydrine	C_7_H_13_NO_2_	18.21	115,244
Sucrose	C_12_H_22_O_11_	9.11	5988
(+)-Magnoflorine	C_20_H_24_NO_4_	5.82	73,337
Nobiletin	C_21_H_22_O_8_	5.67	72,344
2-Methyl-L-proline	C_5_H_9_NO_2_	5.56	6,993,664
Quinicacid	C_7_H_12_O_6_	5.55	6508
Sinensetin	C_20_H_20_O_7_	4.55	145,659
Isoforsythiaside	C_29_H_36_O_15_	3.77	23,958,169
Hesperidin	C_28_H_34_O_15_	3.49	10,621
Magnolol	C_18_H_18_O_2_	3.13	72,300
PurpureasideC	C_35_H_46_O_20_	2.77	13,962,917
6-Demethoxytangeretin	C_19_H_18_O_6_	2.36	629,964
Citricacid	C_6_H_8_O_7_	2.27	124,519,562
Narirutin	C_27_H_32_O_14_	2.22	442,431
Tangeretin	C_20_H_20_O_7_	1.17	68,077
Isosinensetin	C_20_H_20_O_7_	1.15	11,953,944
Cryptochlorogenicacid	C_16_H_18_O_9_	1.10	124,136,972
ViceninII	C_27_H_30_O_15_	1.05	442,664
Betaine	C_5_H_11_NO_2_	1.05	247
L-Phenylalanine	C_9_H_11_NO_2_	1.03	6140
Emodin-8-glucoside	C_21_H_20_O_10_	1.02	99,649
Emodin	C_15_H_10_O_5_	0.99	3220
Gallicacid	C_7_H_6_O_5_	0.93	370
(+)-Catechinhydrate	C_15_H_14_O_6_	0.91	107,957
Rhein	C_15_H_8_O_6_	0.83	10,168
ForsythosideE	C_20_H_30_O_12_	0.82	76,005,395
5-Hydroxymethylfurfural	C_6_H_6_O_3_	0.80	237,332
Hesperetin	C_16_H_14_O_6_	0.73	72,281
Adenine	C_5_H_5_N_5_	0.66	190
EleutherosideB/Syringin	C_17_H_24_O_9_	0.63	5,316,860

### Prediction of action targets of MORRD therapy for SPSI

**Fig. 3:**
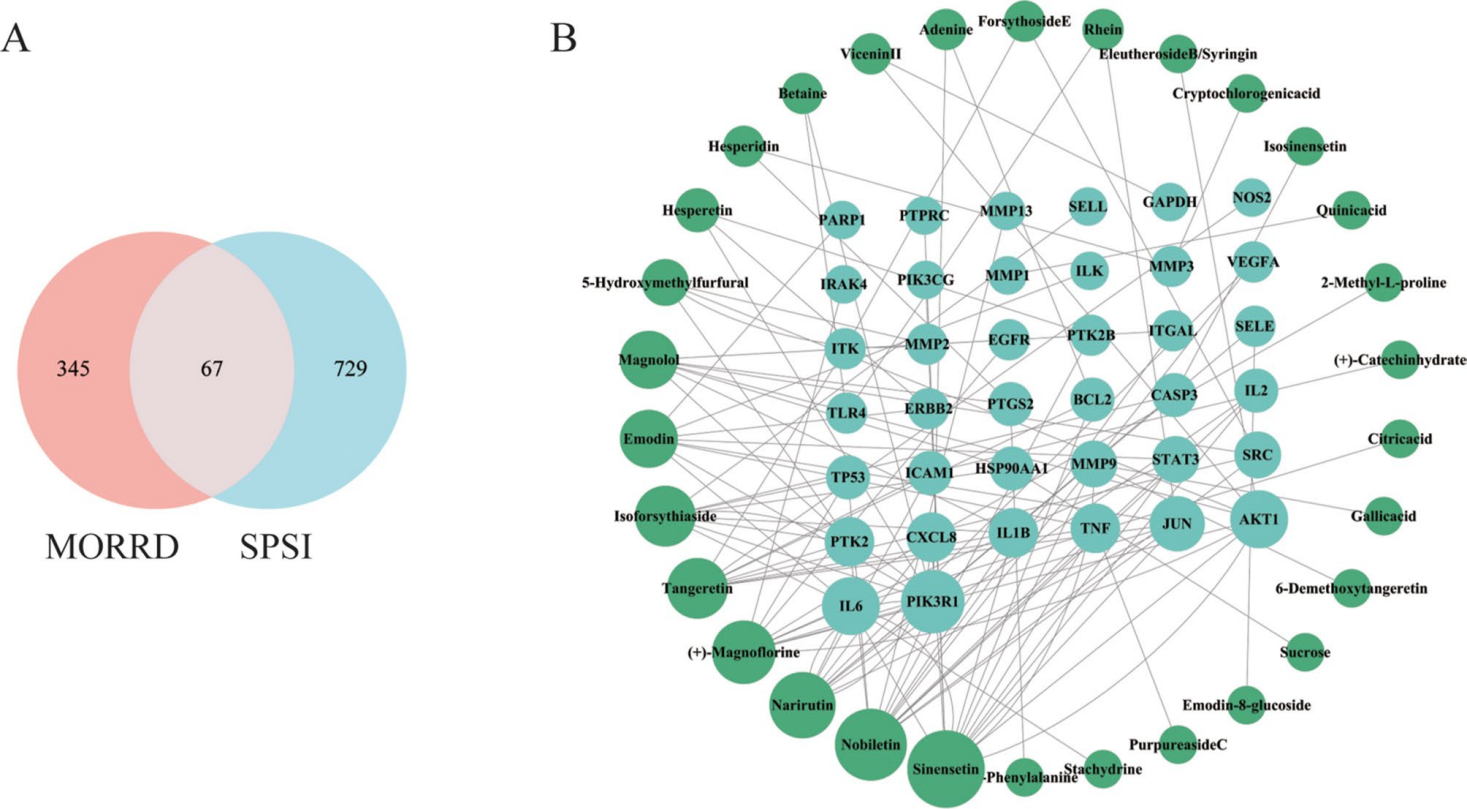
Screening of Active Components of MORRD. **A**. Venn diagram showing the overlap between SPSI-related targets and MORRD component targets. **B**. Network diagram of MORRD active compounds and disease-related targets

SPSI Treatment 260 targets were downloaded from the OMIM database, while 542 targets related to SPSI were obtained from the GeneCards database. A total of 796 distinct targets linked to SPSI were identified after merging and removing duplicate targets. Sixty-seven overlapping targets were found when a Venn diagram was created to determine the possible therapeutic targets of MORRD against SPSI (Fig. 3A).

### Building the network of compound-target interactions

The potential targets of MORRD against SPSI, along with their corresponding active compounds and herbal components, were imported into Cytoscape 3.9.1 to create a compound-target-pathway interaction network. This was done to further investigate the therapeutic mechanisms of MORRD in treating SPSI. The network has 852 edges and 195 nodes, as shown in Fig. 3B. To identify important active chemicals and targets, the topological properties of the nodes were computed using the Analyze Network plugin. Sinensetin, Nobiletin, Narirutin, (+)-Magnoflorine, Tangeretin, Isoforsythiaside, Emodin, and Magnolol were the top eight compounds based on degree value. Among these, Emodin, Magnolol, and (+)-Magnoflorine possess antibacterial properties that directly inhibit the growth of microorganisms causing skin infections, including *Streptococcus* species and *Staphylococcus aureus*(Guo et al. 2021; Li et al. 2023). By reducing the synthesis of inflammatory cytokines, Nobiletin, Tangeretin, and Sinensetin significantly contribute to anti-inflammatory responses and reduce tissue damage caused by skin infections. Magnolol and Isoforsythiaside exhibit antioxidant properties that protect skin cells from oxidative damage induced by infection and promote the restoration of the skin barrier(Park et al. 2023). These findings suggest that MORRD employs a multi-component, multi-target strategy to achieve therapeutic effects against SPSI.

### Protein–protein interaction (PPI) network construction and core target screening

**Fig. 4:**
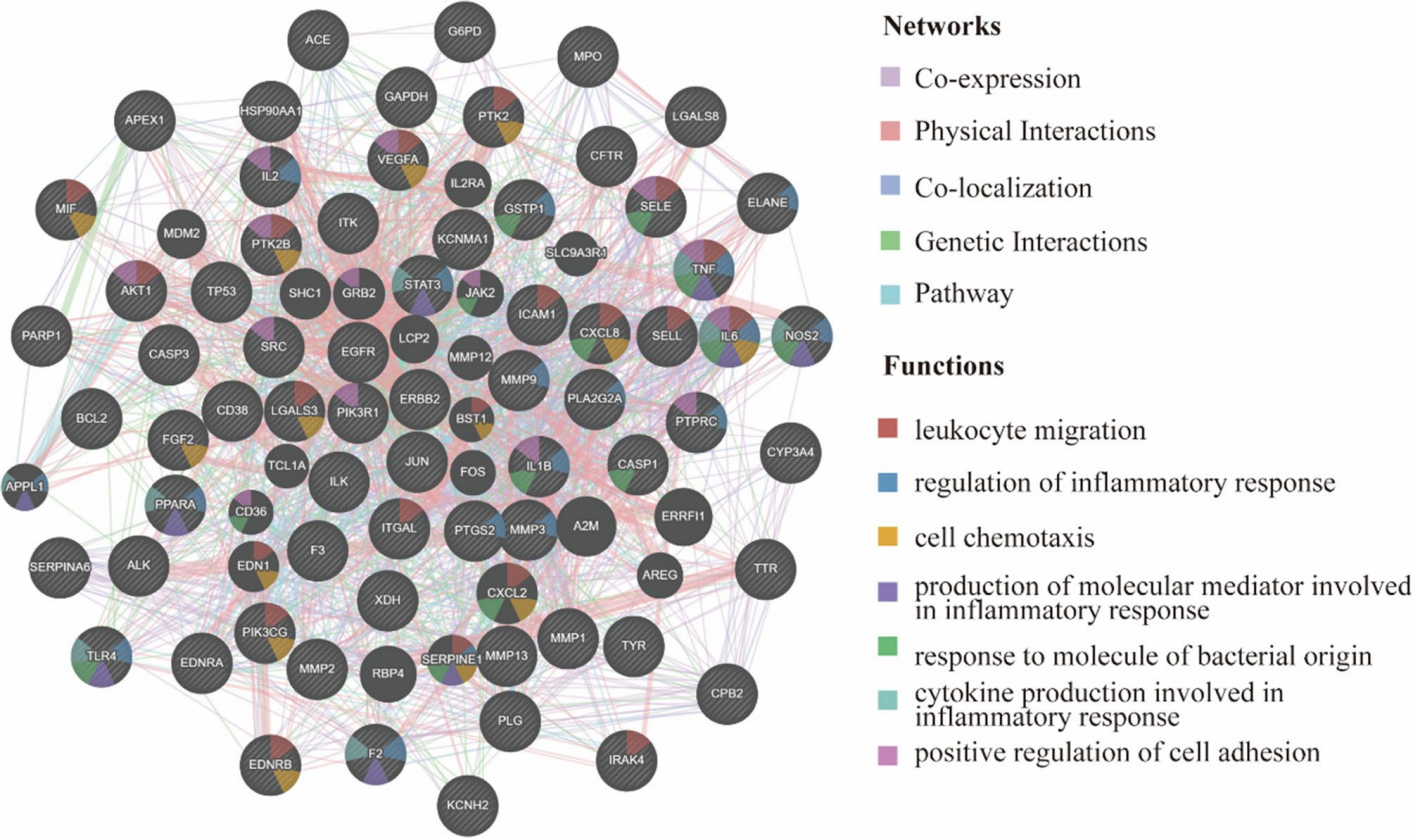
PPI network diagram of the targets of MORRD in the treatment of SPSI

The 67 overlapping targets were imported into the GeneMANIA database to create a protein–protein interaction (PPI) network, as illustrated in Fig. 4. The findings indicated that leukocyte migration and inflammatory response regulation, cell Chemotaxis, the production of cytokines related to the inflammatory response, the production of molecular mediators associated with inflammation, and the positive regulation of cell adhesion are the primary enriched pathways of the network targets. By managing inflammation, regulating immune responses, and directing immune cell migration, these pathways are crucial in the development of skin infections(Pasparakis et al. 2014). When combined, they ensure that the immune system can respond to infections swiftly and effectively while preventing tissue damage and excessive reactions. Altering these pathways could enhance immune responses, reduce the severity and duration of infections, and thereby provide potential therapeutic options for treating skin infections.

### Functional and KEGG pathway

**Fig. 5:**
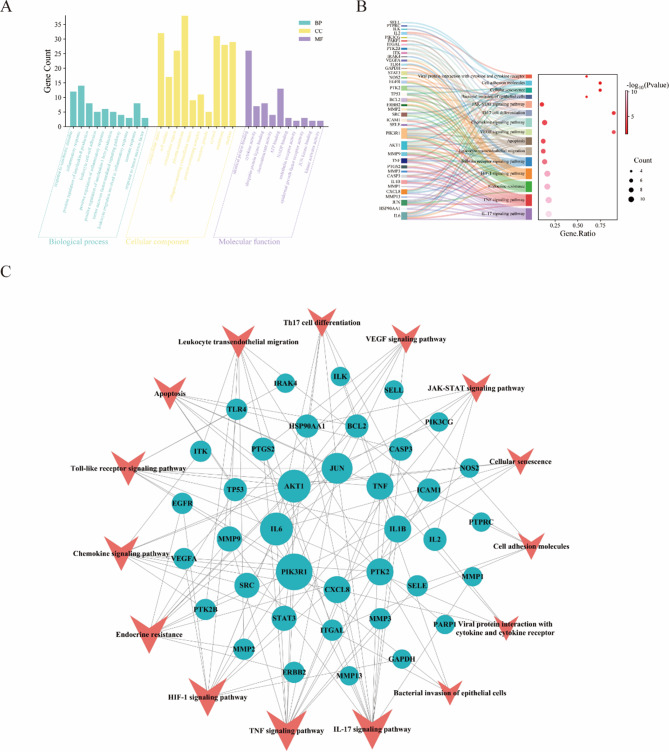
GO and KEGG Pathway Enrichment Analyses of 67 Overlapping Targets. **A**. GO enrichment analysis. **B**. KEGG pathway enrichment analysis. **C**. Intersection targeting points in the KEGG pathway map

Enrichment Analysis of Gene Ontology (GO) Kyoto Encyclopedia of Genes and Genomes (KEGG) and Gene Ontology (GO) enrichment studies were used to determine the metabolic pathways and biological processes that MORRD may impact in the management of SPSI. Following the enrichment analysis of 67 overlapping genes, 1,723 entries were obtained, comprising 402 biological process (BP) terms, 42 cellular component (CC) terms, and 81 molecular function (MF) terms. These targets may be involved in immune cell activation, migration, cytokine modulation, and interactions between cell surfaces and the extracellular matrix, as indicated by the enriched pathways in Fig. 5A. These pathways provide fresh insights for future research and are essential for understanding the immune response, inflammation regulation, and skin infection repair processes.

Out of the 123 pathways identified by KEGG enrichment analysis, 15 exemplary pathways were selected for display in Fig. 5B. The KEGG pathways are primarily categorized into the following groups: human diseases, cellular processes, organismal systems, metabolism, genetic information processing, and environmental information processing. The analysis highlights how skin infections are affected by immunological response, inflammatory response, cell migration, and cell death. Many of these pathways are involved in tissue repair, cytokine release, immune cell activation, and localization—all of which are essential for the immune system’s defense against skin infections.

### Core target screening

The CentiScaPe plugin was utilized to conduct a topological analysis on the identified target proteins and KEGG pathways. The screening criteria included Degree (undirected) > 32.280, Betweenness Centrality (undirected) > 71.059, and Closeness Centrality (undirected) > 0.006. Figure 5C displays the resulting network. As the primary targets of MORRD in the treatment of SPSI, the research identified eight core targets with high degree values in the network: PIK3R1, IL6, AKT1, JUN, TNF, IL1B, PTK2, and CXCL8(Figure 5C; Table 2).

**Table 2:** Core target information

Gene Name	ID	Resolution	Description	Function
PIK3R1	5GJI	0.9 A	Phosphotyrosine Phosphatidylinositol 3-kinase regulatory subunit alpha	PIK3R1 is a regulatory subunit of PI3K, which is involved in activating the AKT pathway and regulating the ability to phagocytose bacteria
IL-6	1ALU	1.9 A	Interleukin-6 N-linked(GlcNAc…) asparagine	IL-6, a multifunctional cytokine, regulates immunity and boosts neutrophil bactericidal activity
AKT1	1UNQ	0.98 A	N6-acetyllysine RAC-alpha serine/threonine-protein kinase	AKT1 can affect autophagy pathways (such as mTOR signaling), indirectly regulating the ability of cells to clear bacteria
JUN	6Y3V	1.5 A	Sumoylated lysine Phosphothreonine; by PAK2	JUN affects macrophage polarization and promotes antibacterial effects such as phagocytic function, ROS generation, and NO synthesis
TNF	5UUI	1.4 A	Cytoplasmic Helical; Signal-anchor for type II membrane protein	TNF can induce the expression of antimicrobial effector factors such as NO (nitric oxide) and ROS (reactive oxygen species), enhancing pathogen clearance
IL-1β	8RYS	1.16 A	Interleukin-1 beta Removed in mature form; by CASP1	It is one of the core regulatory factors of inflammatory response after bacterial infection
PTK2	6YOJ	1.36 A	N-acetylalanine Focal adhesion kinase 1	Immune cells such as neutrophils and macrophages rely on PTK2 regulation to enhance bacterial clearance
CXCL8	4XDX	0.95 A	MDNCF-a Interleukin-8	CXCL8, a potent neutrophil chemokine, binds CXCR1/2 to direct their migration to bacterial infection sites

### Molecular docking

**Fig. 6:**
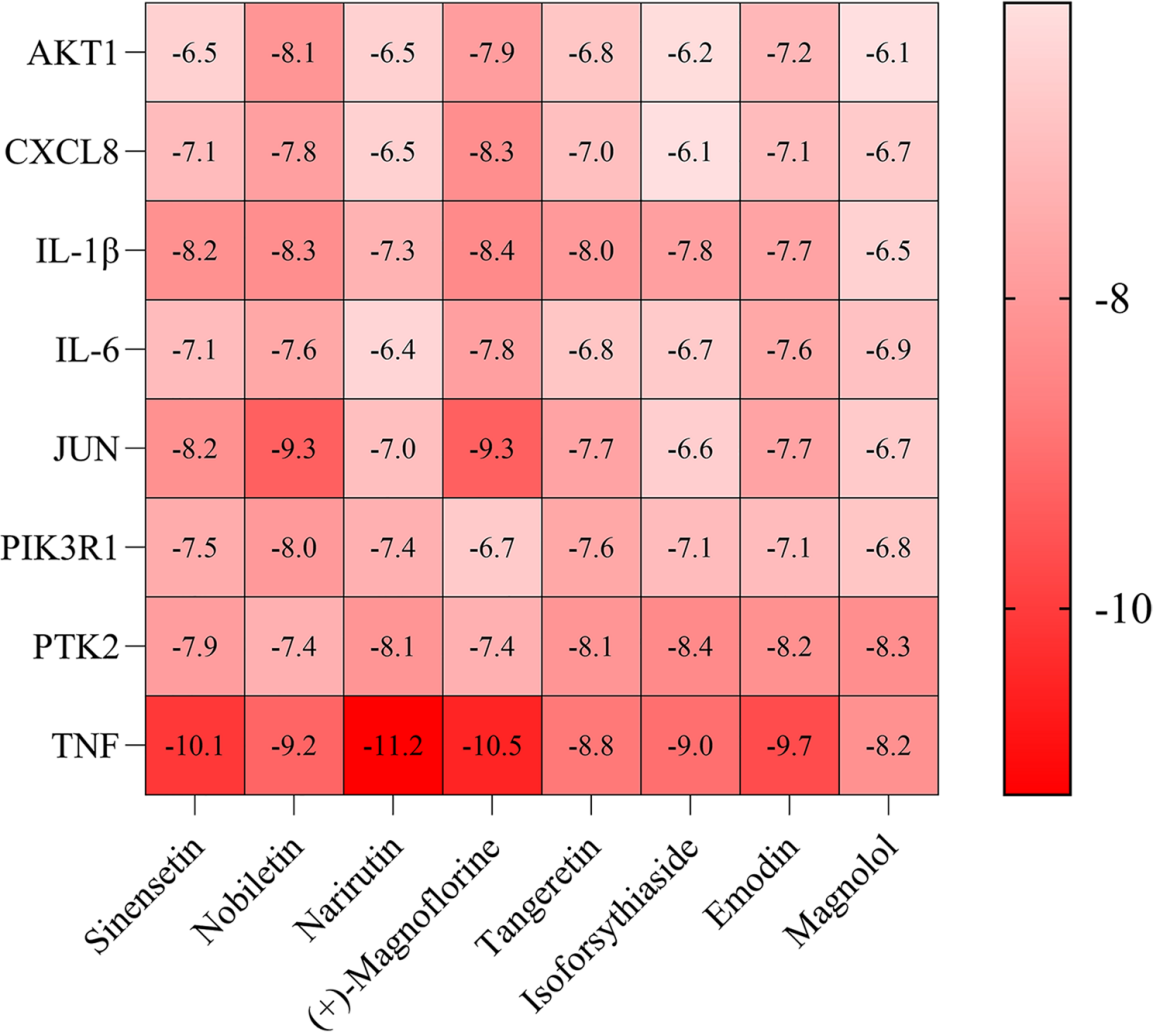
Heatmap of the binding energy (kcal/mol) of key targets and active compounds

PIK3R1 (UniProt ID: 5GJI), IL6 (UniProt ID: 1ALU), AKT1 (UniProt ID: 1UNQ), JUN (UniProt ID: 6Y3V), TNF (UniProt ID: 5UUI), IL1-β (UniProt ID: 8RYS), PTK2 (UniProt ID: 6YOJ), and CXCL8 (UniProt ID: 4XDX) were the core target proteins selected for molecular docking. The top eight active compounds associated with the most targets were docked with these proteins. Figure 6 shows the binding energies (kcal/mol) obtained from the docking analysis. A lower binding energy value indicates a stronger binding affinity. Binding scores below − 5.0 kcal/mol suggest moderate affinity, while those below − 7.0 kcal/mol indicate high affinity. Eight ligand-receptor pairs exhibited binding energies below − 9.0 kcal/mol, confirming that all ligand-receptor docking binding energies were under 0 kcal/mol. Binding energy is a crucial metric for assessing the degree of interaction between substances and their target proteins; higher negative values signify stronger binding and suggest a compound’s ability to effectively alter the target’s function(Arcon et al. 2017). The results indicate that TNF, IL-1β, and JUN are likely to be significant targets in managing SPSI.

**Fig. 7:**
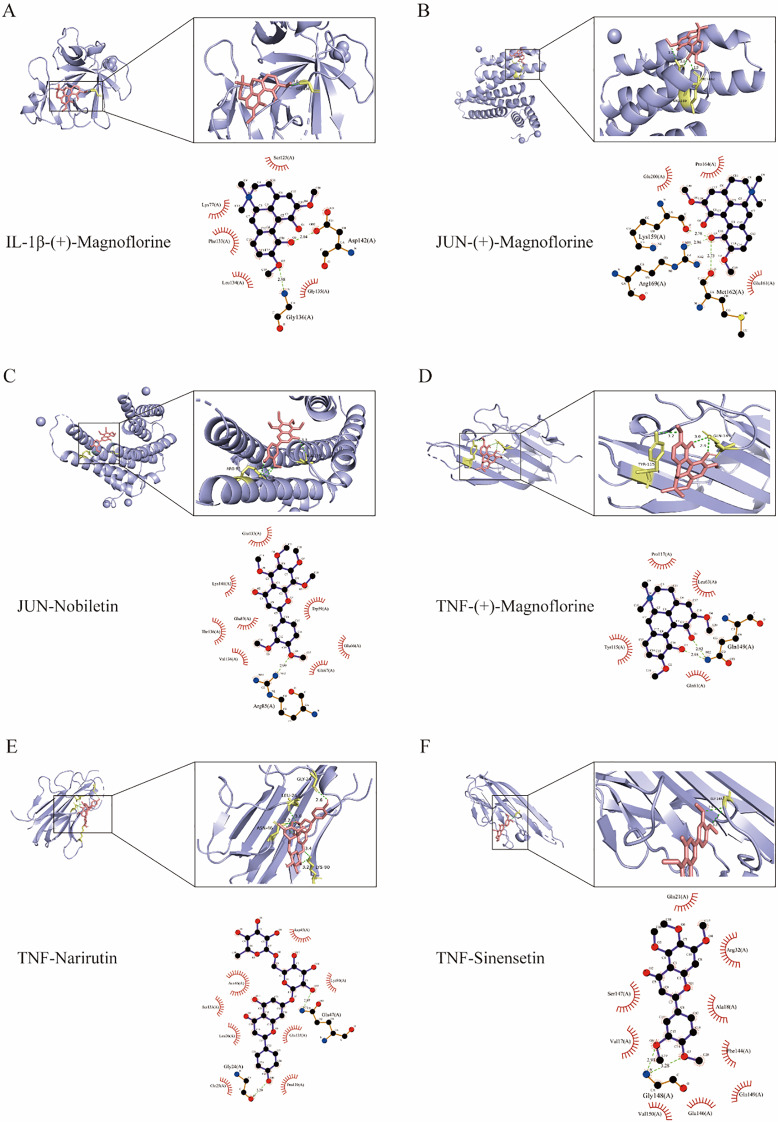
Molecular Docking Visualization of Core Targets with Active Compounds. **A**. IL1β–(+)-Magnoflorine. **B**. JUN–(+)-Magnoflorine. **C**. JUN–Nobiletin. **D**. TNF–(+)-Magnoflorine. **E**. TNF–Narirutin. **F**. TNF–Sinensetin

The compound-target complexes with high binding affinities and their binding processes were visualized using PyMOL 3. The two- and three-dimensional molecular docking diagrams of the main targets containing active compounds are shown in Fig. 7.

### In vitro verification of MORRD in the treatment of SPSI

**Fig. 8:**
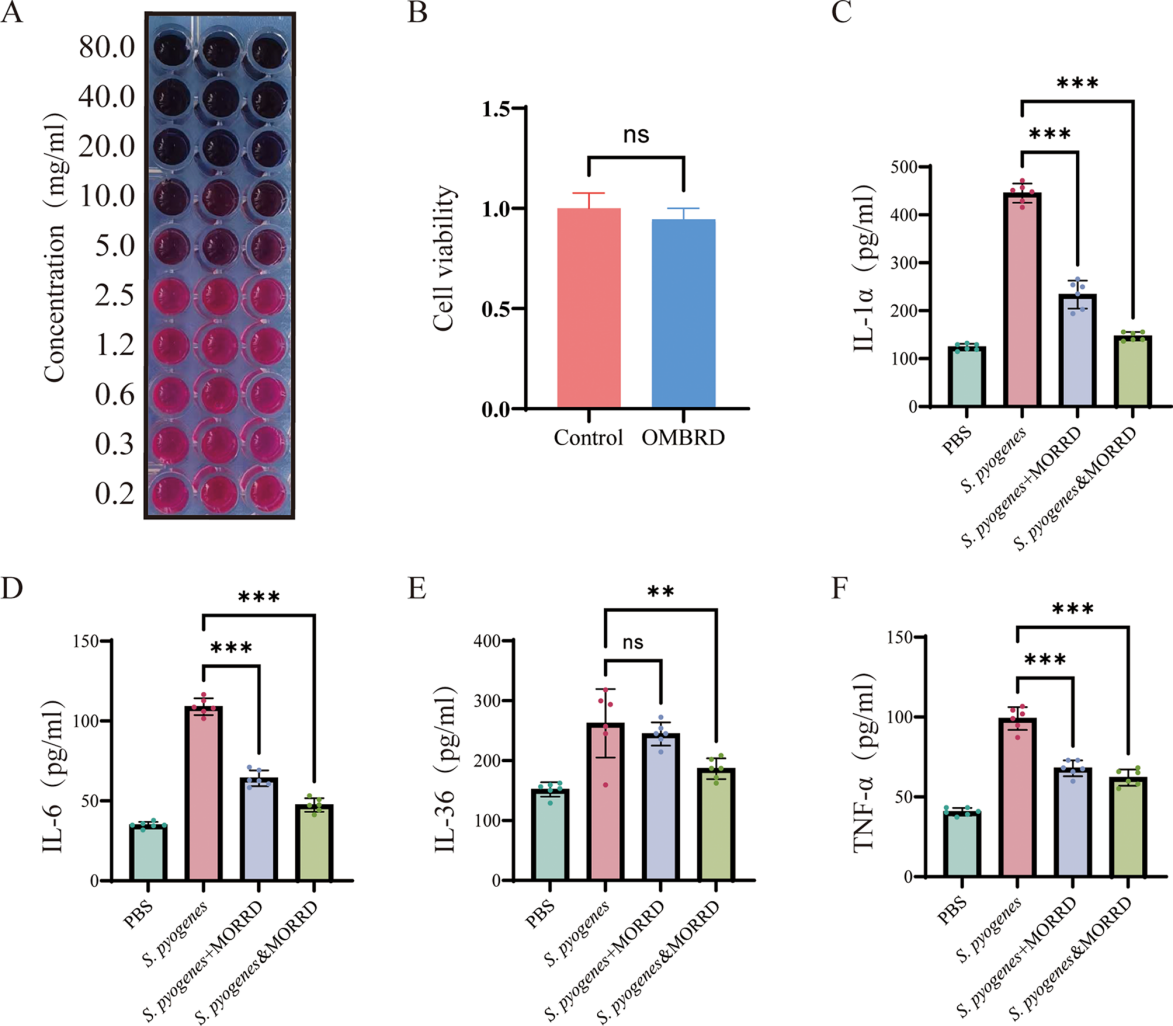
In vitro validation of MORRD treatment for SPSI. **A**. MORRD MIC determination. **B**. Cell Viability Assay. **C**-**F**. ELISA assessment of changes in IL-1α, IL-6, TNF-α, and IL-36 levels in HaCaT cells after 3 h of *S. pyogenes* invasion. PBS: control group; *S. pyogenes*: *S. pyogenes* invasion for 3 h, followed by PBS washing and 21 h of continued culture; *S. pyogenes* + MORRD: *S. pyogenes* invasion for 3 h, followed by PBS washing and culture with MORRD added to the medium for 21 h; *S. pyogenes* & MORRD: simultaneous co-infection with *S. pyogenes* and MORRD for 3 h, followed by PBS washing and 21 h of continued culture. ^*^, ^**^, and ^***^ indicate statistical significance at ^*^P ≤ 0.05, ^**^P ≤ 0.01, and ^***^P ≤ 0.001, respectively; ns = not significant. *n* = 6

Human epidermal keratinocytes (HaCaT cells) were cultivated and subjected to bacterial invasion tests to confirm the therapeutic impact of MORRD on SPSI. The expression levels of inflammatory cytokines TNF-α, IL-1α, IL-6, and IL-36 were measured to evaluate the antibacterial activity of MORRD. Specifically, four groups were created from the experimental model: Group 1: 100 µL of PBS was added, and the mixture was incubated for three hours. Group 2: Invasion group: After three hours of infection with 1 × 10^6^ CFU of bacterial, cells were washed three times with PBS, and 100 µL of culture media was added. Group 3: Post-invasion treatment group: Cells were treated with a combination of 50 µL culture medium and 50 µL MORRD after three hours of bacterial invasion and three PBS washes. Group 4: Co-incubation group: *S. pyogenes* and MORRD were added to a human skin-equivalent model simultaneously. They co-incubated for three hours, after which they were washed three times with PBS and received 100 µL of culture media. According to the results, the post-invasion therapy group and the co-incubation group exhibited significantly lower levels of inflammatory cytokines than the invasion group. These results indicate that MORRD has therapeutic effects after bacterial invasion and suppresses the proliferation of *S. pyogenes* in keratinocytes (Fig. 8, Supplementary Fig. 1).

## Discussion

*S. pyogenes* is a well-known human-specific pathogen that causes more than 100 million skin infections and 600 million pharyngeal infections each year. This bacterium is commonly found in nature, human and animal excrement, and the nasopharynx of healthy individuals (Hurst et al. 2022). It is a Gram-positive coccus that is spherical or ovoid in shape, measuring 0.6–1 μm in diameter, and is typically grouped in chains. It can adhere to and colonize skin cells due to several virulence factors and strong adhesion capabilities. By breaking down connective tissue, its hyaluronidase production facilitates the spread of infection. Common clinical signs of *S. pyogenes* infection include folliculitis, furuncles, abscesses, acute cellulitis, erysipelas, and acute lymphangitis. Symptoms often involve skin redness, swelling, discomfort, induration, elevated local temperature, and tenderness. Severe cases may present with variability, necrosis, ulceration, and functional impairment. Severe infections can lead to sepsis and bacteremia, and some patients may also experience systemic symptoms like fever, headache, and malaise (Siggins et al. 2020). Despite extensive research on invasive disease, many aspects of the bacterium’s normal colonization of the skin and nasopharynx remain unknown. Antibiotics are often used in clinical settings to treat infections caused by pathogens such as *Staphylococcus aureus* and *S. pyogenes*; however, the increasing prevalence of bacterial resistance to macrolides due to the widespread use of antibiotics underscores the urgent need for new treatment agents.

Among the many benefits of TCM are its broad pharmacological action, low toxicity, multi-component, multi-target effects, and reduced susceptibility to resistance development. For our antibacterial tests, we used aqueous extracts of *Aurantii Fructus Immaturus* (AF), *Magnolia officinalis* (MO), and *Rheum rhabarbarum* L. (RR) in this investigation. To the best of our knowledge, this is the first study demonstrating that MORRD’s active ingredients inhibit the growth of *S. pyogenes* and treat skin diseases caused by this pathogen. TCM theory states that MORRD’s main purpose is to facilitate the flow of qi, with MO serving as the primary herb. RR eliminates heat and promotes bowel movements, Zhishi (*Aurantii Fructus Immaturus*) alleviates qi stagnation and addresses accumulation, and MO alleviates fullness and stimulates qi circulation. These three herbs work in concert to support defecation and qi flow. This formula was initially documented in Zhang Zhongjing’s Jin Kui Yao Lue at the beginning of the third century CE. Recent pharmacological research shows that the active ingredients of these three plants possess antibacterial properties (Guo et al. 2021; Müller-Heupt et al. 2022; Gao et al. 2023). The MO is derived from the dried bark and root bark of *Magnolia officinalis* or *Magnolia officinalis* var. biloba (Magnoliaceae), according to Zhong Guo Yao Dian. Its properties include drying dampness, resolving phlegm, descending qi, and easing fullness. It is characterized by its heated and bitter qualities and addresses symptoms such as epigastric and abdominal distension. on by moisture, food retention, and stagnation of qi. It also enters the lung meridian to treat cough and asthma with Excessive phlegm enters the spleen, stomach, and large intestine meridians. The MO contains anti-inflammatory, analgesic, anticancer, and antioxidant properties, according to modern pharmacology. RR is an annual herbaceous plant with thick, large, yellow roots that belongs to the Polygonaceae family’s genus Rumex. Globally, there are more than 200 species, most of which are found in North America and Europe (Pliszko 2019). Since it is cold and bitter, RR effectively removes heat, prevents buildup, increases blood flow, halts bleeding, and possesses antibacterial, anticoagulant, and anti-inflammatory properties. The RR “promotes renewal by removing old and harmful substances”, according to Xue Zheng Lun, and it acts on blood diseases to restore harmony. The AF, which is rich in flavonoids and the alkaloid synephrine, is primarily utilized to treat constipation, food stagnation, and qi stagnation accompanied by phlegm obstruction. There are 19 prescriptions for AF in the Shang Han Zang Bing Lun. The active ingredients in AF, according to current pharmacological research, relieve intestinal dryness and constipation, promote gastrointestinal motility, speed up metabolism, and exhibit antimicrobial and lipid metabolism-regulating properties. In conclusion, MORRD’s three active ingredients are all antibacterial and anti-inflammatory. We employed network pharmacology techniques to clarify the mechanisms behind MORRD’s antibacterial and anti-infective properties. Our study identified 101 overlapping targets between SPSI-related targets and MORRD’s active components, indicating potential interactions between SPSI and MORRD components.

This study identified several biological pathways and molecular mechanisms that may be implicated in skin infections through an integrated analysis of data from the Kyoto Encyclopedia of Genes and Genomes (KEGG) and Gene Ontology (GO). GO analysis clarified the roles of target proteins in biological processes, cellular components, and molecular activities, while KEGG analysis provided further insight into their involvement in specific signaling pathways. Collectively, these results suggest that the chemicals under investigation may modulate several crucial biological and immunological pathways, thus playing significant roles in immune response, inflammatory regulation, cell repair, and antibacterial activity. GO enrichment revealed substantial participation in immunological response, inflammatory response, cell migration, and immune cell regulation-including cytokine generation, leukocyte migration, and inflammation. According to KEGG pathway analysis, these targets are primarily enriched in key signaling pathways such as the VEGF, TNF, chemokine, apoptotic, and IL-17 pathways. Notably, skin infections are largely caused by the IL-17 and TNF pathways (Moos et al. 2023; Reiss et al. 2023). TNF is crucial for modulating immune cell activity and local inflammation, while IL-17 is a key regulator of immunological and inflammatory responses, promoting the recruitment and activation of immune cells. The pathways of chemokine signaling and leukocyte transendothelial migration highlight how immune cells quickly relocate to infection sites to support antibacterial defense (Zhu et al. 2024). The significance of aging and cell death in immune control is underscored by the enrichment of apoptosis and cellular senescence Pathways may affect the number and function of immune cells while avoiding excessive immune-mediated tissue damage (Padilha et al. 2022). These findings suggest that controlling these pathways may successfully improve immune responses, reduce excessive inflammation, encourage tissue regeneration, and enhance antibacterial activity.

The connections between various natural chemicals and immune-related targets were further uncovered by binding energy heatmap analysis, highlighting their crucial roles in antibacterial activity. The use of natural remedies for skin infections involves direct pathogen removal, as well as immune modulation and inflammation reduction. Potential antibacterial processes are clarified by the quantitative insights into the intensity of the interaction between important targets and drugs provided by the binding energy data. Natural substances exert antibacterial effects primarily in two ways: by directly preventing pathogen growth and by enhancing host defense through immunological modulation. Emodin is known for its broad-spectrum antibacterial activity, which directly hinders bacterial growth by rupturing bacterial cell membranes and preventing DNA synthesis (Urgesa et al. 2024). Therefore, both immunomodulatory and direct antimicrobial actions may contribute to its antibacterial effects. Isoforsythiaside and magnolol prevent bacterial proliferation by altering their metabolic pathways. Magnolol shows significant binding affinity with PIK3R1, implying that it may inhibit pathogen growth by regulating metabolic pathways associated with bacteria. Natural chemicals often demonstrate multi-target effects by simultaneously interacting with various targets and altering different signaling pathways, potentially increasing their antibacterial efficiency in complex pathogenic environments. By binding several immune regulatory targets, including CXCL8 and IL-6, sinensetin may enhance the recruitment and activation of immune cells, improving the effectiveness of the immune response. Further enhancing its antibacterial action, sinensetin may also prevent bacterial invasion and growth (Ge et al. 2024). When taken as a whole, these natural substances reduce excessive inflammation, promote the migration of immune cells, and assist in bacterial removal, providing new therapeutic approaches and methods for treating skin infections. Their remarkable therapeutic potential is underscored by their broad-spectrum antibacterial action and ability to influence various biological systems.

We conducted in vitro tests to confirm MORRD’s therapeutic efficacy against SPSI and its inhibitory effect on *S. pyogenes*. In 96-well plates, MORRD broth was serially diluted twice. A final bacterial suspension concentration of approximately 5 × 10^5^ CFU/mL was achieved by selecting morphologically similar single colonies from solid media and cultivating them in liquid media at 37 °C with shaking at 220 rpm for 4–6 h until reaching the logarithmic growth phase. MORRD was found to have a minimum inhibitory concentration (MIC) of 20 mg/mL. The findings indicate that all three individual components—*Magnolia officinalis*, *Rheum rhabarbarum* L., and *Aurantii Fructus Immaturus*—exhibit measurable antibacterial activity when tested separately. However, when combined within the complete MORRD formulation, their inhibitory effect was significantly enhanced, demonstrating the strongest antibacterial response among all tested groups(Supplementary Figs. 2, 3). This observation strongly suggests a synergistic interaction among the components, rather than a merely additive effect(Caesar and Cech 2019). Such synergy implies that the combined formulation may target multiple bacterial pathways or enhance membrane permeability, thereby increasing overall efficacy. These results underscore the potential of rational multi-component combinations in developing more effective antibacterial therapies and provide a foundation for further mechanistic studies on the interactions between these herbal constituents.In response to bacterial infection, keratinocytes commonly release interleukins, including IL-1α, IL-6, IL-36, and TNF-α. Significant suppression of bacterial growth was observed when *S. pyogenes* was inoculated into HaCaT cells in the presence of MORRD. Moreover, compared to untreated controls, MORRD therapy resulted in a substantial reduction in interleukin expression after three hours of infection. Tissue damage often arises from excessive inflammation and cytokine release, which are indicators of immune system activation in skin infections. By modulating immune factors and diminishing local inflammatory responses, our in vitro tests not only validated MORRD’s inhibitory effects on *S. pyogenes* but also suggested its potential therapeutic use for treating skin infections. These findings support MORRD’s promise as an anti-inflammatory and antibacterial agent. This study is the first to investigate MORRD’s impact on SPSI by integrating network pharmacology analysis with experimental validation. It is important to note that the quality of database information significantly affects the accuracy and reliability of target projections. Initial in vitro tests were performed to address this limitation, and the results indicated that MORRD has therapeutic effects against SPSI and limits its proliferation. However, further experimental research is necessary to elucidate the specific chemical pathways responsible for these benefits.

We carried out in vitro tests to confirm MORRD’s inhibitory action on *S. pyogenes* and its therapeutic effectiveness against SPSI. In 96-well plates, MORRD broth was serially diluted twice. Single colonies with comparable morphologies were chosen from solid media and moved into liquid culture medium. After 4–6 h of shaking at 220 rpm and 37 °C, the cultures reached the logarithmic growth phase, yielding a bacterial suspension concentration of roughly 5 × 10^5^ CFU/mL. MORRD was found to have a minimum inhibitory concentration (MIC) of 20 mg/mL, which significantly inhibited *S. pyogenes* growth at this concentration.

The HaCaT keratinocyte model was then used to examine the immunomodulatory effects of MORRD during *S. pyogenes* infection. Common interleukins released by keratinocytes during bacterial infections include IL-1α, IL-6, IL-36, and TNF-α. These interleukins are crucial for immunological and inflammatory reactions in skin infections. *S. pyogenes* infected HaCaT cells, and MORRD was introduced while the infection was going on. In comparison to the untreated positive control group, the results showed that MORRD dramatically decreased interleukin levels and hindered *S. pyogenes* growth after three hours of infection. When the immune system is activated during a skin infection, inflammation and increased cytokine release are common side effects that can cause tissue damage. This trial showed MORRD’s potential as a treatment for skin infections in addition to confirming its inhibitory action on *S. pyogenes*. MORRD reduced local inflammation brought on by *S. pyogenes* by modifying immune factor levels. According to these results, MORRD not only successfully suppresses the growth of *S. pyogenes* but also controls the immunological response that the bacteria induce by lowering the release of inflammatory cytokines. This offers compelling evidence for its potential as a medicinal agent with antibacterial and anti-inflammatory properties.

This study is the first to investigate the therapeutic effects of MORRD on SPSI by integrating experimental validation with network pharmacology analysis. Although UHPLC-MS/MS successfully identified the active components of MORRD and their potential targets associated with SPSI, the reliability of the analysis and predictions largely depends on the quality and completeness of the data.

To address this limitation, we conducted preliminary in vitro experiments, which demonstrated MORRD’s inhibitory effect on S. pyogenes and its therapeutic potential against related skin infections. However, further experimental studies are required to elucidate the underlying mechanisms in greater detail.

Collectively, these findings provide both a theoretical foundation and essential experimental evidence supporting the potential clinical application of MORRD. They also lay the groundwork for future research into its antibacterial and anti-inflammatory properties in the treatment of skin infections.

## Conclusion

This study is the first to confirm that the traditional Chinese medicine compound MORRD effectively inhibits *S. pyogenes* (MIC = 20 mg/mL) and significantly alleviates the skin infections it causes through a multi-target synergistic mechanism. Mechanistic studies demonstrate that the active ingredients in MORRD (such as Sinensetin, Nobiletin, and (+)-Magnoflorine) regulate key immune pathways like IL-17/TNF/chemokines, suppress the secretion of pro-inflammatory factors (IL-1α/IL-6/IL-36/TNF-α), and directly disrupt bacterial cell membranes and metabolic processes, achieving a dual “antibacterial-anti-inflammatory” effect. This provides a novel multi-target therapeutic strategy for combating drug-resistant *S. pyogenes* infections.

## Materials and methods

### Preparation of MORRD

Fifteen grams of *Magnolia officinalis* (MO), 18 g of *Rheum rhabarbarum* L. (RR), and 9 g of *Aurantii Fructus Immaturus* (AF) were added to 1 L of water and boiled for three hours. The decoction was then allowed to cool to room temperature before being freeze-dried for 18 h. The resulting powder was dissolved in sterile water to a final concentration of 80 mg/mL and stored for future use.

### UHPLC-MS/MS analysis

After thorough mixing, an appropriate amount of sample was transferred into a 15 mL centrifuge tube, to which 10 mL of 50% methanol aqueous solution (v/v, methanol: water = 50:50) was added. The mixture was sonicated for 30 min. Subsequently, 1 mL of the supernatant was transferred into another centrifuge tube and centrifuged at 14,000 rpm for 5 min. The resulting supernatant was filtered through a 0.22 μm microporous membrane and transferred into an injection vial. Chromatographic conditions were as follows: column, ACQUITY UPLC HSS T3 (2.1 × 100 mm, 1.8 μm); column temperature, 35 °C; injection volume, 10 µL; flow rate, 0.3 mL/min; mobile phase A, deionized water containing 0.1% formic acid; mobile phase B, acetonitrile containing 0.1% formic acid; gradient elution. The detailed gradient elution program is as follows:

**Table:** 

Time	FlowRate(mL/min)	%A	%B
0	0.3	100	0
10	0.3	70	30
25	0.3	60	40
30	0.3	50	50
40	0.3	30	70
45	0.3	0	100
60	0.3	0	100
60.5	0.3	100	0
70	0.3	100	0

Mass spectrometry data were acquired using a Q Exactive Orbitrap high-resolution mass spectrometer. The detection mode was Full MS-ddMS² with separate scans in positive and negative ion modes. The scan range was m/z 100–1200. The MS¹ resolution was set to 70,000, while the MS² resolution was 17,500. The ion source voltage was 3.2 kV, the capillary temperature was 320 °C, the auxiliary gas heater temperature was 350 °C, the sheath gas flow rate was 40 L/min, and the auxiliary gas flow rate was 15 L/min. The AGC target was set to 1e6, and TopN was 5. The collision energy for triggering MS² scans was a stepped normalized collision energy (NCE) at 30, 40, and 50 (Ferreira, Queiroz and Analysis 2021; Toja-Camba et al. 2024; Spolnik et al. 2018).

### Identification of active components and potential targets of MORRD

Raw mass spectrometry data were processed using Compound Discoverer 3.2 software for feature peak extraction, elemental matching, molecular formula prediction, and isotopic distribution matching, with mass error tolerance set within 5 ppm. Feature peaks were identified by comparing them against the mzCloud online database and an in-house mzVault traditional Chinese medicine natural products database. Positive identifications met the criteria of mass error < 5 ppm, consistent isotopic distribution, and mzVault best match score > 70. After manual verification and removal of redundant results, active compounds of MORRD were obtained. SMILES strings of active compounds were retrieved from the PubChem database (https://pubchem.ncbi.nlm.nih.gov/) and imported into the SwissTargetPrediction platform (http://www.swisstargetprediction.ch/), with the species set to༂Homo sapiens༂and a probability threshold > 0 for potential target prediction.

### Construction of SPSI-related target dataset

SPSI-related targets were retrieved by searching the terms༂*Streptococcus pyogenes* skin infection༂in the GeneCards (https://www.genecards.org/) and Online Mendelian Inheritance in Man (OMIM, https://mirror.omim.org/) databases. The results from both databases were merged, and duplicates were removed to create a comprehensive SPSI-related target database. The resulting targets were intersected with MORRD active compound targets using Venny 2.1 (https://bioinfogp.cnb.csic.es/tools/venny/index.html/), yielding overlapping targets for MORRD treatment of SPSI. These overlapping targets, along with MORRD active compounds, were imported into Cytoscape 3.9.1 software, where the top eight core active compounds were screened based on degree values.

### Protein–protein interaction (PPI) network construction and core target screening

The overlapping targets were uploaded to the GeneMANIA platform (https://genemania.org/) to construct the protein–protein interaction (PPI) network. The species was set to༂Homo sapiens༂, with the default parameters unchanged. The PPI network model was obtained accordingly (Cai et al. 2023).

### Gene ontology (GO) and Kyoto encyclopedia of genes and genomes (KEGG) enrichment analysis

Core targets were imported into the DAVID database (https://david.ncifcrf.gov/)29 for GO and KEGG pathway enrichment analysis, using *P* < 0.05 as the significance threshold (Li et al. 2021). Enrichment results were visualized using the Wei Sheng Xin platform (https://www.bioinformatics.com.cn/). GO enrichment included categories of biological process (BP), cellular component (CC), and molecular function (MF). KEGG pathway data were imported into Cytoscape 3.9.1, and eight core targets were selected based on degree values.

### Molecular docking

The target protein 3D structure pattern “PDB” format file and the active ingredient 2D structure pattern “MOL2” format file were obtained from the PDB database (http://www.rcsb.org/), UniProt database (https://www.uniprot.org/) and TCMSP (https://tcmsp-e.com/) using Chem3D and PyMOL3 The active ingredients are individually optimized for structural conversion and energy optimization. Using AutoDockTools 1. 5. 7 Molecular docking, first the receptor protein and ligand small molecules are dehydrated and hydrogenated, and then the core components completed by the above treatment are molecularly docked with the 3D structure of the core target, Auto Dock Vina is used for molecular docking calculation, and the result with the lowest binding energy is selected as the best conformation according to the receptor-ligand binding energy, and the binding energy heat map is drawn with Oringin2021, and visualized with PyMOL3 and LigPlus (Liu et al. 2022).

### Cell culture

Human keratinocyte HaCaT cells were obtained from Shanghai Jinyuan Biological Technology Co., Ltd. HaCaT cells were maintained in DMEM medium enriched with 10% fetal bovine serum (FBS) and 1% penicillin–streptomycin solution at 37 °C in a humidified environment containing 5% CO₂ (Yang et al. 2023; Zhang et al. 2024).

### *S. pyogenes* culture

*S. pyogenes* strain ATCC BAA-947 was obtained from Fuxiang Biotechnology. The bacteria were cultured in Todd-Hewitt broth enriched with 0.2% yeast extract (THY broth) at 37 °C (Song et al. 2020).

### Minimum inhibitory concentration (MIC) determination

*S. pyogenes* was first cultured to the logarithmic growth phase, and the cell density was preliminarily estimated by measuring the optical density at 600 nm (OD₆₀₀). The suspension was then serially diluted and plated onto agar plates. After incubation at 37 °C for 18 h, colony-forming units (CFUs) were counted. Based on the plate count results, the bacterial suspension was adjusted to 1 × 10⁶ CFU/mL. A volume of 100 µL of the bacterial suspension was added to each well, followed by 100 µL of the test sample, resulting in a final bacterial concentration of 5 × 10⁵ CFU/mL per well. The microtitre plates were incubated at 37 °C for 18 h. MIC was visually observed and recorded as the lowest concentration of the test sample that inhibited visible bacterial growth, using 20 µL of resazurin solution as a viability indicator (Nwabor et al. 2023).

### Drug toxicity test

Cells were divided into two groups: the experimental group received 20 mg/mL of MORRD, while the control group was treated with an equal volume of PBS. Cell viability was evaluated using the CCK-8 assay to assess the cytotoxicity of the treatment(Hu et al. 2023).

### ELISA

After three hours of *S. pyogenes* invasion of human skin equivalents, supernatants were collected and centrifuged at 1,000 × g for ten minutes to remove particulates and aggregates. Samples were then incubated sequentially with antibody working solutions, washed five times (with residual moisture blotted dry during washes), and followed by incubation with substrate solution. After adding the stop solution and thorough mixing, optical density (OD) at 600 nm was measured to detect changes in IL-1α, IL-6, TNF-α, and IL-36 levels. ELISA kits were purchased from MEIMIAN Biological Technology Co., Ltd.

### Statistical analysis

Experimental results were based on averages from at least three technical or biological replicates. Statistical analyses and graphing were conducted using GraphPad Prism 9.0 software(Guo et al. 2024). Differences were deemed statistically significant at *P* ≤ 0.05.

## Supplementary Information

Below is the link to the electronic supplementary material.

Supplementary Material 1

Supplementary Material 2

## Data Availability

The datasets used during the current study are available from the corresponding author on reasonable request.

## Abbreviations

AF: Aurantii Fructus Immaturus
GAS: Group A Streptococci
GO: Gene Ontology
IL-1 α: Interleukin-1 α
IL-36: Interleukin-36
IL-6: Interleukin-6
KEGG: Kyoto Encyclopedia of Genes and Genomes
MIC: Minimum Inhibitory Concentration
MO: Magnolia officinalis
MORRD: Magnolia officinalis Rheum rhabarbarum Decoction
RR: Rheum rhabarbarum L
SPSI: Streptococcus pyogenes skin infection
S. pyogenes: Streptococcus pyogenes
TCM: Traditional Chinese medicine
TIC: Total Ion Chromatograms
TNF- α: Tumor necrosis factor- α
WHO: World Health Organization
