# Supplementary material for: Utilizing network pharmacology and other tools to examine active components and mechanism of action of Magnolia officinalis rheum rhabarbarum decoction in treating Streptococcus pyogenes skin infections
Source: Bioresour Bioprocess. 2025 Aug 26;12(1):92. doi: 10.1186/s40643-025-00933-1 (PMC12381315; doi:10.1186/s40643-025-00933-1)
Supplement: Supplementary file 2 — Supplementary Material 2 [file 40643_2025_933_MOESM2_ESM.docx]

1.The minimum inhibitory concentration (MIC) of MORRD against *Streptococcus pyogenes* was determined using the broth microdilution method in accordance with CLSI guidelines. The assay was performed in 96-well microtiter plates and included three groups: Negative control: wells containing only THY medium without any antibacterial agent, used to confirm the normal growth of bacteria. Experimental group: wells treated with the MORRD formulation to assess its antibacterial activity.Positive control: wells containing gentamicin, a broad-spectrum antibiotic, used as a reference for effective inhibition.

Bacterial suspensions were prepared to a final concentration of 5 × 10⁵ CFU/mL per well. After adding the respective treatments, plates were incubated at 37°C for 18 hours. Bacterial viability was evaluated using 20 μL of resazurin solution as an indicator. The MIC was defined as the lowest concentration that prevented visible color change (indicating bacterial growth).

The results of this assay are shown in Supplementary Fig 1, which clearly illustrates the comparative inhibitory effects of MORRD and gentamicin, validating the experimental setup and supporting the antibacterial potential of MORRD.


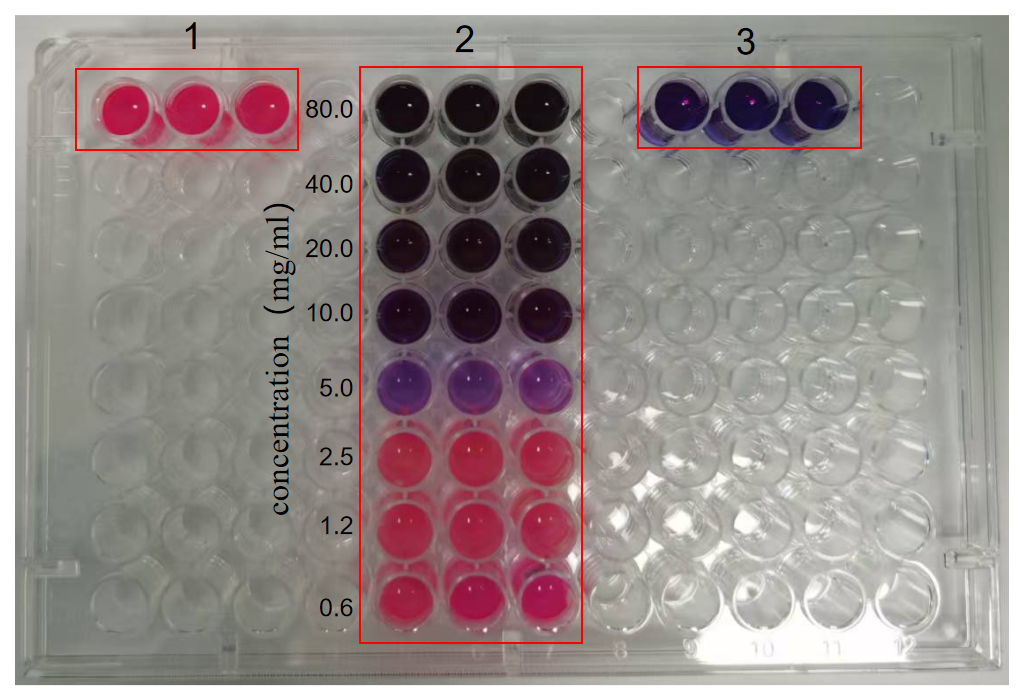


Supplementary Fig.1 *Streptococcus pyogenes* MIC assay. 1. Negative control:THY medium. 2. Experimental group:MORRD. 3. Positive control: gentamicin.

2.We conducted antibacterial activity testing on the three individual components of MORRD (Officinal Magnolia Bark, Rhubarb, and Aurantii Fructus Immaturus) as shown in Supplementary Fig 2, as well as on their pairwise combinations, shown in Supplementary Fig 3: (A) Officinal Magnolia Bark + Rhubarb, (B) Officinal Magnolia Bark + Aurantii Fructus Immaturus, and (C) Rhubarb + Aurantii Fructus Immaturus. These results were systematically compared with the full MORRD formulation.

The findings indicate that all three individual components exhibit antibacterial activity, and their combination within the complete MORRD formulation produces the strongest inhibitory effect, suggesting a synergistic interaction among them.


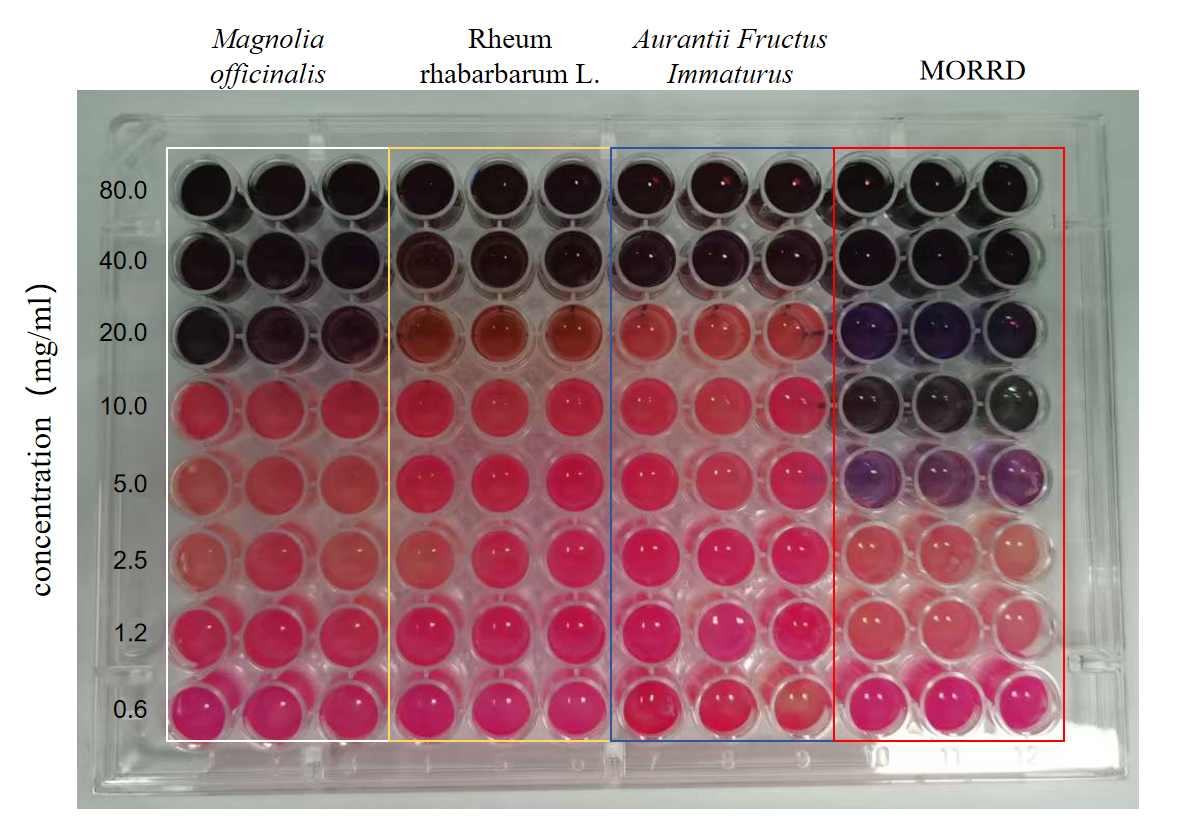


Supplementary Fig.2 MORRD is more inhibitive of Streptococcus pyogenes than its single component.


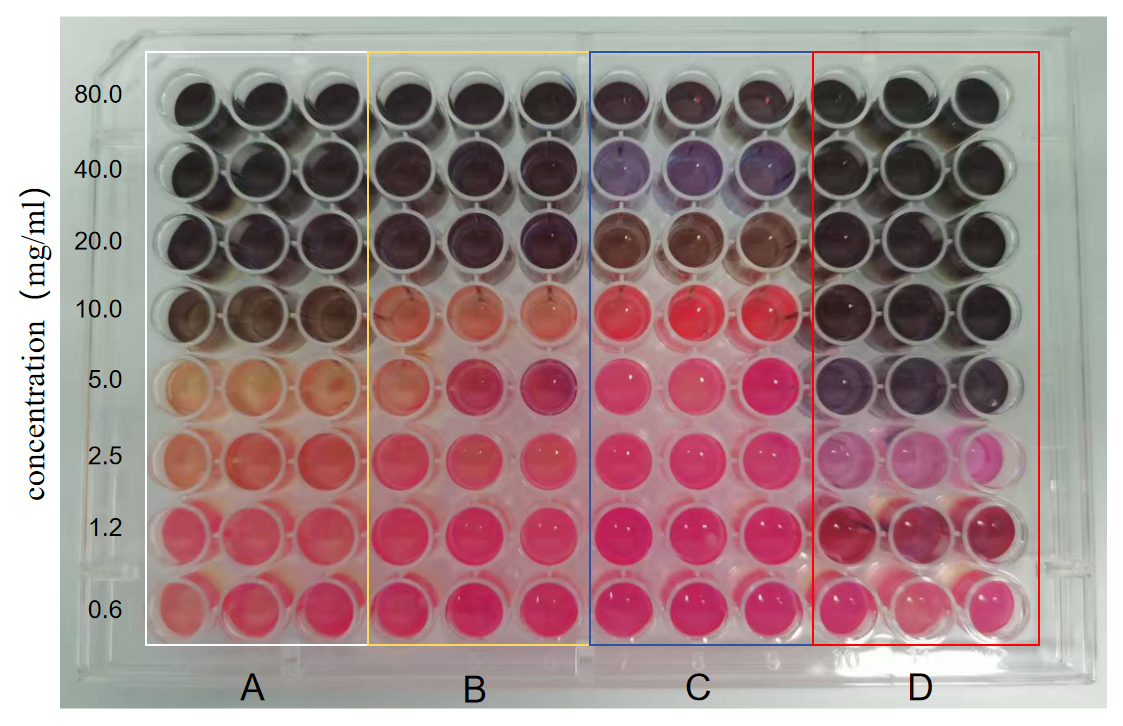


Supplementary Fig.3 Pairwise combination testing of two drug components for antibacterial activity. A. *Magnolia officinalis*+*Rheum rhabarbarum* L. B. *Magnolia officinalis*+*Aurantii Fructus Immaturus.* C. *Rheum rhabarbarum* L.+*Aurantii Fructus Immaturus*
